# Association of Soluble IL-1 Receptor Type 2 with Recovery of Left Ventricular Function and Clinical Outcomes in Acute Myocardial Infarction

**DOI:** 10.31083/j.rcm2311372

**Published:** 2022-10-31

**Authors:** Sui-Feng Liu, Song Liu, Qiao-Ting Yu, Tang-Gang Gao, Yang Zhang, Jia-Yi Cai, Chun-Wen Jia, Ya-Nan Zhao, Feng Gao

**Affiliations:** ^1^Department of Cardiology, Zhongshan Hospital of Xiamen University, School of Medicine, Xiamen University, 361000 Xiamen, Fujian, China; ^2^Central Clinical Laboratory, Zhongshan Hospital of Xiamen University, School of Medicine, Xiamen University, 361000 Xiamen, Fujian, China

**Keywords:** acute myocardial infarction, inflammation, interleukin-1 receptor type 2

## Abstract

**Background:**

The role of soluble interleukin-1 receptor type 2 (sIL-1R2) in acute myocardial infarction (AMI) remains undocumented. In the present study, we aimed to evaluate the possible associations of sIL-1R2 with left ventricular (LV) function, remodeling and future clinical events in the setting of AMI.

**Methods:**

Circulating sIL-1R2 levels were quantified after percutaneous coronary intervention (PCI) on day 1 of hospital admission for 204 AMI patients, and upon enrollment of 204 healthy controls. Echocardiography was conducted in the acute phase and at 12-month follow-up. Adverse clinical events were registered after 12 months.

**Results:**

Circulating sIL-1R2 levels were significantly higher in AMI patients than in healthy controls (medians respectively 6652.81 pg/mL, 3799.13 pg/mL, *p *< 0.0001). AMI patients with sIL-1R2 levels less than the median had a larger proportion of worsened LV ejection fraction [a decrease in LV ejection fraction (LVEF) of more than 10% units] and reduced LVEF (a final LVEF <50%). After multivariate adjustment, sIL-1R2 levels less than the median were associated with an increased risk of worsened LVEF [odds ratio (OR): 3.7, 95% confidence interval (CI): 1.6–8.5, *p* = 0.002] and reduced LVEF at 12 months (OR: 2.1, 95% CI: 1.1–4.3, *p* = 0.035). Moreover, low sIL-1R2 levels were associated with an increased risk of having an adverse clinical event during the first 12 months after AMI [hazard ratio (HR): 2.5, 95% CI: 1.0–6.1, *p* = 0.039].

**Conclusions:**

Low levels of circulating sIL-1R2 were associated with impaired recovery of LV function and adverse clinical outcomes in AMI patients. These findings might contribute to understanding the important role of sIL-1R2 in postinfarction inflammation.

## 1. Introduction

Acute myocardial infarction (AMI) is a leading contributor to morbidity and mortality worldwide [1]. Inflammation plays a pivotal role in the development of atherosclerotic plaques, as well as acceleration of plaque rupture and local thrombosis [2]. Inflammation is a double-edged sword. Although the post-AMI inflammatory response is prerequisite for normal healing of damaged heart tissue, excessive inflammation is associated with maladaptive left ventricular (LV) remodeling, progressive heart failure, and ultimately adverse clinical outcomes [3]. Thus, inflammation in AMI has potential as a therapeutic target.

**Fig. 1.**
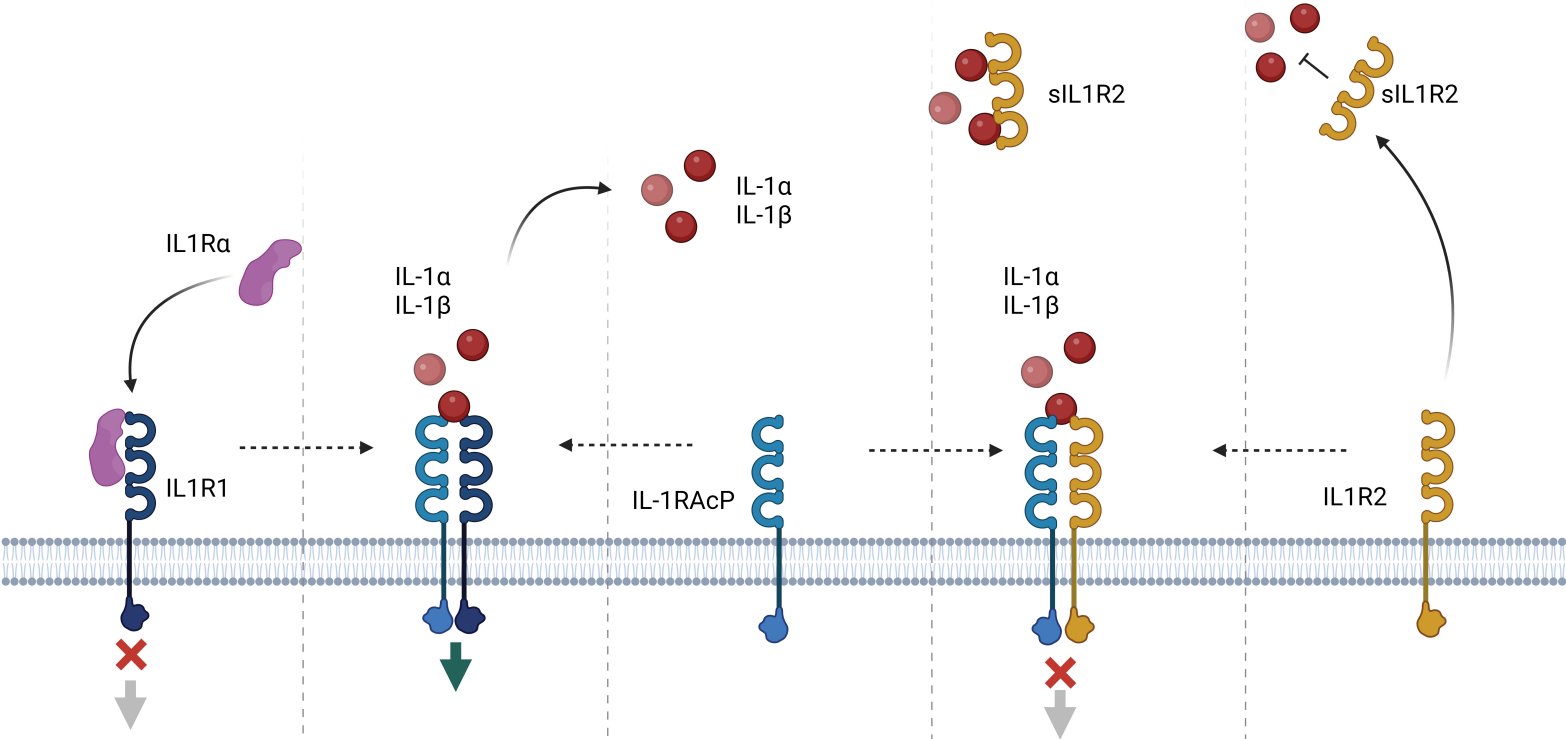
**The IL-1 system. Activation**. The main extracellular soluble activators IL-1α and IL-1β bind to IL-1R1. IL-1RAcP is necessary for the formation of the signal transduction complexes. *Inhibition*. IL-1Rα prevents IL-1 from interacting with IL-1R1. Membrane IL-1R2 acts as a decoy receptor binding to IL-1, but not initiating signaling. Soluble IL-1R2 exhibits anti-inflammatory activity by sequestering IL-1 in the circulation. IL, interleukin; R, receptor; AcP, accessory protein; s, soluble. *Figure created with https://BioRender.com*.

Interleukin-1 (IL-1) plays a central role as a mediator propagating the inflammatory response and is considered the main target in atherosclerotic thromboprotection [4]. Two proteins, IL-1α and IL-1β, induce potent inflammatory responses [5]. IL-1 receptor antagonists (IL-1Ra) and IL-1 receptor type 2 (IL-1R2) are separate mechanisms for inhibiting IL-1-mediated inflammation [6]. The binding of IL-1 to IL-1 receptor type 1 (IL-1R1) is blocked by IL-1Ra. IL-1R2 acts as a decoy receptor on the cell surface or in a soluble form (sIL-1R2) in the circulation, inhibiting the IL-1-mediated inflammatory response (Fig. 1) [7]. Maintaining a balance between agonist and antagonist levels avoids exaggerated inflammatory responses. Recently, the Canakinumab Anti-inflammatory Thrombosis Outcomes Study (CANTOS) showed that blocking inflammation with the anti-IL-1β monoclonal antibody canakinumab reduced heart attacks, strokes and new-onset diabetes among patients with prior myocardial infarction (MI) [8]. Other clinical trials have shown that the IL-1 receptor antagonist anakinra exhibits anti-inflammatory properties in patients with MI [9, 10]. However, little is known about the levels of sIL-1R2, which significantly affect net activity in IL-1-related pathways in the setting of AMI.

We hypothesize that sIL-1R2 may be an inflammatory indicator associated with LV remodeling after AMI. Using blood sampling and repeated echocardiography, we aim to assess the possible associations between sIL-1R2 and LVEF, ventricular remodeling and adverse clinical events.

## 2. Materials and Methods

### 2.1 Patients and Study Design 

**Fig. 2.**
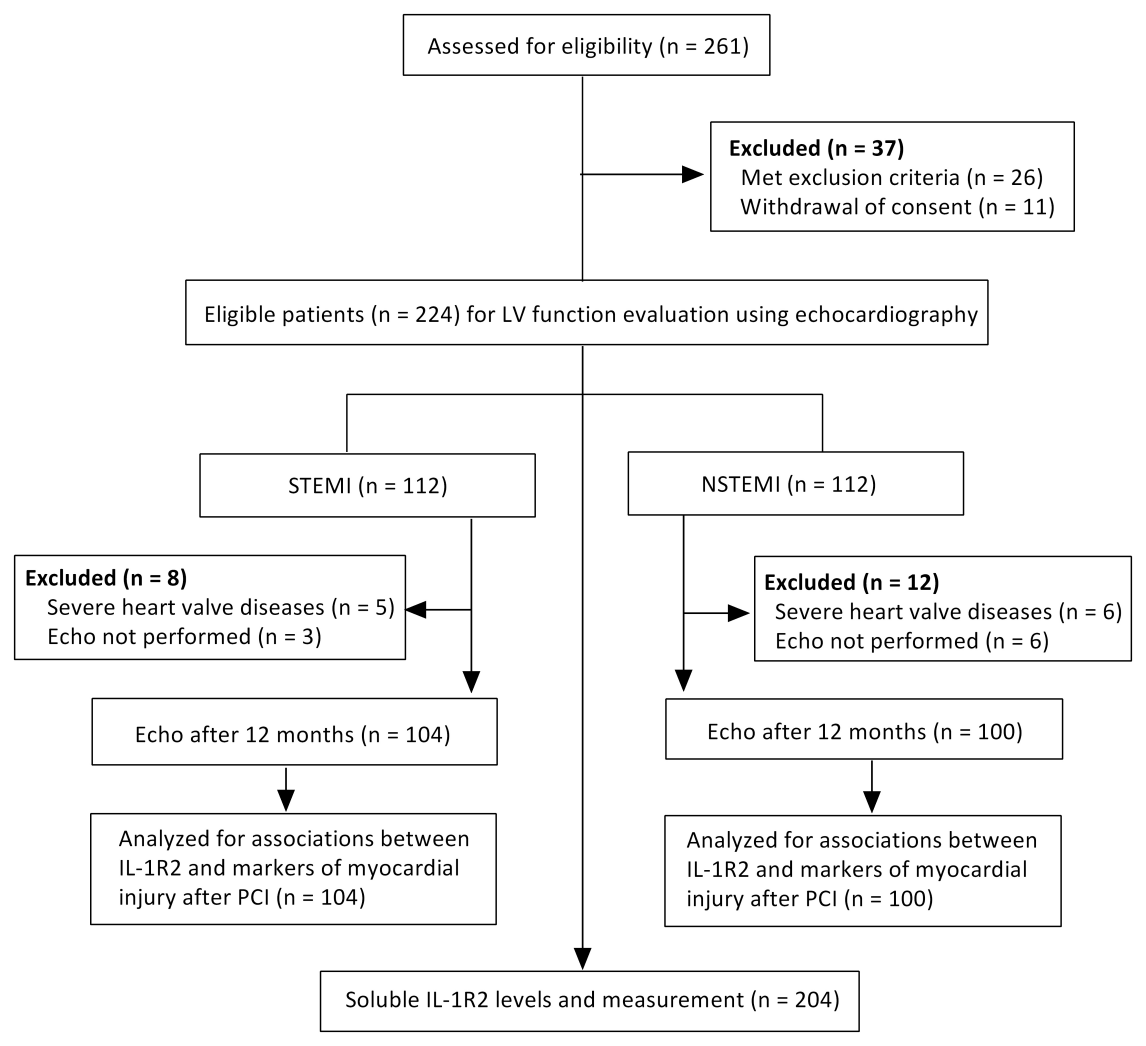
**Study flow diagram**. Echo, echocardiography; PCI, percutaneous coronary intervention.

Patients with AMI symptom duration <12 h, including non-ST-segment elevation myocardial infarction (NSTEMI) and ST-segment elevation myocardial infarction (STEMI), were recruited between June 2020 and July 2021 at the Department of Cardiology, Zhongshan Hospital of Xiamen University, China (n = 261). Patients were included in the presence of changed cardiac biomarkers, typical symptoms and representative electrocardiographic changes according to current guidelines [11, 12, 13]. Exclusion criteria were previous history of AMI, clinically unstable status (cardiac arrest, cardiogenic shock, hypotension, or pulmonary congestion), atrial fibrillation, severe heart valve disease, renal failure (serum creatinine ˃200 μmol/L), severe hepatic diseases, severe peripheral vascular disease, cerebrovascular event in past three months, obesity, tumors, various acute and chronic infectious diseases, autoimmune diseases and other serious illnesses that may interfere with the study results, or withdrawal of informed consent (Fig. 2). A total of 204 AMI patients were retained after exclusions. Echocardiography was performed in the acute phase following the percutaneous coronary intervention (PCI) procedure and repeated after 12 months to assess LV function. Adverse clinical events were registered at a median follow-up of 12 months after the index infarction. Clinical end points were defined as heart failure, reinfarction, stroke or death. We screened 310 individuals with no signs or symptoms of cardiovascular disease (CVD) from the Department of Cardiology’s outpatient registry during the same period, selecting 204 age- and sex-matched individuals as healthy controls. To minimize the effect of metabolic diseases on sIL-1R2 levels, we further divided controls into those with and without metabolic diseases. Metabolic diseases referred to overweight/obesity (BMI ≥25 kg/$m^{2}$), diabetes and metabolic syndrome. Metabolic syndrome was defined according to the World Health Organization criteria [14].

### 2.2 Blood Preparation and Measurement

Blood samples for the analysis of sIL-1R2 were drawn from healthy controls (n = 204) upon enrollment and from AMI patients at day 1 following the PCI procedure, median 23.8 h after the onset of AMI (n = 204). All samples were centrifuged at 4000 rpm for 10 min at 4 °C to obtain plasma samples and then stored at –80 °C pending further analysis.

To determine plasma levels of sIL-1R2, we used human IL-1R2 enzymelinked immunosorbent assay (ELISA) kits from R&D Systems (DY263, Minneapolis, MN, USA). Levels of N-terminal pro-B-type natriuretic peptide (NT-proBNP) on hospital admission were measured using an enzyme immunoassay (Roche Diagnostics, Mannheim, Germany), while high-sensitivity cardiac troponin T (TnT) was determined on an Elecsys 2010 analyzer (also from Roche Diagnostics). C-reactive protein (CRP) tests and other routine biochemical analyses were performed by routine laboratory assays daily or every other day. The maximum values of CRP and TnT measured during hospitalization were defined as the peak CRP and the peak TnT, respectively. The intra- and inter-assay coefficients of variation were <10% for all assays.

### 2.3 Echocardiography Analysis

We performed echocardiography within 2 days after PCI and after 12 months using a Vivid E9 scanner with a phased-array transducer (M5S) (GE Ultrasound, Horten, Norway). Endocardial boundaries were outlined in the four-chamber and two-chamber sections to calculate the volume parameters, including LV end-systolic volume (LVESV) and left ventricular end-diastolic volume (LVEDV). The biplane Simpson method was adopted to calculate LVEF. Echocardiographic images were collected by 2 experienced radiologists who were unaware of the patient’s clinical data. Radiologists checked each other and made decisions together to improve diagnostic consistency.

### 2.4 Statistical Analysis

The Kolmogorov-Smirnov test was used to analyze the distribution of data. For continuous variables, the median (interquartile range) was used for statistical description, and the Mann-Whitney U test and Kruskal-Wallis tests were used for intergroup comparison. Categorical variables were described in the form of counts (%), and their intergroup comparisons were assessed by the chi-square test. Associations between sIL-1R2 and clinical variables were tested by Spearman’s correlation. Levels of sIL-1R2 were analyzed in logistic regression analyses with adverse LV remodeling, worsened LVEF and reduced LVEF as binary responses. Adverse LV remodeling was defined as LV dilatation (LVEDV increase of >20% or LVESV increase of >15%) [15]. A worsened LVEF was defined as a decrease in LVEF >10% [16] and a reduced LVEF as a LVEF of <50% after 12 months [17]. Baseline variables that were considered clinically relevant or that showed an association with sIL-1R2 with a *p*-value < 0.05 were entered into the logistic regression model. Continuous variables with skewed distributions including TnT, CRP, NT-proBNP, neutrophils, neutrophil to lymphocyte ratio (NLR), neutrophil to platelet ratio (NPR) and platelet to lymphocyte ratio (PLR), were logarithmically transformed. The association between sIL-1R2 and adverse clinical events was evaluated using Cox regression. The number of variables included in the models was restricted because of the relatively low number of events available. The diagnostic performance of sIL-1R2 as a predictor for a composite endpoint of mortality, reinfarction, rehospitalization for heart failure or stroke was evaluated by the area under the receiver operating characteristic curve (AUC). Statistical analyses were performed with SPSS 28.0 (SPSS Inc., Chicago, IL, USA) or STATA 17.0 (StataCorp LP, College Station, TX, USA). A *p*-value < 0.05 was considered statistically significant.

## 3. Results

### 3.1 Clinical and Biochemical Characteristics

A total of 261 AMI patients and 204 healthy controls were evaluated (Fig. 2). Samples from patients were acquired on day 1 after PCI (median 23.8 h after the onset of AMI). Patients were dichotomized by the median expression value for sIL-1R2. Samples from controls were acquired upon enrollment. Clinical characteristics are presented in Table 1. AMI patients had a greater proportion of smokers, drinkers, essential hypertension, hypercholesterolemia and diabetes. Patients with low sIL-1R2 levels had significantly lower admission neutrophil levels than patients with high sIL-1R2 levels.

**Table 1.** ** Clinical characteristics of healthy controls and of patients (total and according to sIL-1R2 levels)**.

		Healthy controls (N = 204)	All Patients (N = 204)	sIL-1R2 ≤Median (≤6652.81 pg/mL)	sIL-1R2 >Median (>6652.81 pg/mL)
Baseline characteristics					
	Age, years	62 (29–92)	62 (32–94)	63 (36–88)	62 (32–94)
	Gender, male, n (%)	157 (77.5)	157 (77.5)	77 (75.5)	80 (79.4)
	BMI, kg/$m^{2}$	24.5 (16.6–31.7)	24.5 (17.6–32.9)	24.3 (18.8–29.4)	24.7 (17.6–32.9)
	Essential hypertension, n (%)	88 (43.1)	107 (53.4)^**^	58 (57.8)	49 (49)
	Hypercholesterolemia, n (%)	28 (13.7)	130 (63.7)^**^	64 (62.7)	66 (64.7)
	Diabetes mellitus, n (%)	25 (12.3)	79 (39.7)^**^	42 (42.1)	37 (37.3)
	Current smoker, n (%)	76 (37.3)	103 (51)^**^	50 (49.0)	53 (52.9)
	Alcohol consumption, n (%)	37 (18.1)	65 (32.4)^**^	32 (31.4)	33 (33.3)
Clinical characteristics					
	STEMI, n (%)	-̶	104 (51.0)	53 (52.0)	51 (50.0)
	Triple vessel lesion, n (%)	-̶	29 (17.3)	14 (17.0)	14 (17.6)
Biochemical analyses					
	Peak troponin T, ng/L	n.d.	3649.4 (126.1–10,000)	3221.8 (126.1–10,000)	4077 (134.0–10,000)
	NT-proBNP, nmol/L	122.3 (5–496)	2747.9 (10–35,000)^**^	3021.1 (23.1–29,571)	2474.7 (10–35,000)
	CRP, mg/L	3.6 (0.2–38.4)	30.1 (0.2–194.2)^**^	28.9 (0.2–194.2)	31.3 (0.4–179.5)
	WBC, ${\mathrm{10}}^{9}$/L	6.8 (3.1–11.9)	10.2 (3.1–24.6)^**^	10.1 (4.1–24.6)	10.4 (3.1–24.6)
	Neutrophil, ${\mathrm{10}}^{9}$/L	4.31 (1.6–8.6)	7.64 (1.8–21.5)^**^	6.9 (3.1–21.5)	8.43 ${\mathrm{(1.8–21.5)}}^{\#}$
	Lymphocyte, ${\mathrm{10}}^{9}$/L	1.9 (0.4–4.1)	1.7 (0.3–5.4)	1.6 (0.3–5.4)	1.7 (0.5–3.5)
	PLT, ${\mathrm{10}}^{9}$/L	230.9 (79–427)	231.3 (25–449)	228.3 (25–422)	234 (77–449)
	NLR	2.7 (0.9–15.7)	6.0 (0.6–32.5)^**^	6.0 (0.6–32.5)	6.1 (1.1–19.8)
	PLR	135.7 (52.2–337.3)	165.7 (23.1–627.7)^**^	171.4 (23.1–627.7)	160 (46.4–542.1)
	NPR	0.019 (0.006–0.092)	0.047 (0.009–0.31)^**^	0.051 (0.01–0.31)	0.044 (0.009–0.088)
	Hemoglobin, g/dL	139.2 (57–175)	133.4 (58–169)	130.7 (58–167)	136 (64–169)
	Creatinine, µmol/L	79.1 (31.5–185.2)	83.6 (38.6–198.7)^**^	82.8 (40.9–194.4)	84.4 (38.6–198.7)
	UA, µmol/L	402.1 (198–578.2)	409.2 (98.7–815)	423.2 (173–760.2)	395.2 (98.7–815)
	TG, mmol/L	2.0 (0.5–8.7)	1.7 (0.4–9.6)	1.6 (0.4–5.9)	1.9 (0.4–9.6)
	TC, mmol/L	4.8 (1.4–10)	5.1 (2.0–11.6)	5.1 (2.0–11.6)	5.1 (2.3–10.2)
	HDL-C, mmol/L	1.2 (0.6–2.1)	1.1 (0.4–2.2)	1.1 (0.4–2.2)	1.1 (0.6–1.8)
	LDL-C, mmol/L	3.1 (0.8–7.4)	3.4 ${\mathrm{(1.2–8.6)}}^{*}$	3.5 (1.2–8.6)	3.4 (1.3–7.4)
	Glucose, mmol/L	6.3 (3.8–11.8)	9.4 (4.2–27.8)^**^	8.9 (4.2–24.4)	9.9 (4.3–27.8)
	HbA1c, %	5.9 (4.8–10.5)	6.7 ${\mathrm{(4.8–17.3)}}^{*}$	6.5 (5.0–11.5)	6.9 (4.8–17.3)

BMI, body mass index; STEMI, ST-segment elevation myocardial infarction; NT-proBNP, N-terminal pro–B-type natriuretic peptide; WBC, white blood cell; CRP, C-reactive protein; NLR, neutrophil-to-lymphocyte ratio; PLR, platelet-to-lymphocyte ratio; NPR, neutrophil-to-platelet ratio; UA, uric acid; TG, triglyceride; TC, total cholesterol; HDL-C, high-density lipoprotein cholesterol; LDL-C, low-density lipoprotein cholesterol; HbA1c, glycosylated hemoglobin; n.d., no data. Values are medians (interquartile ranges), means ± SD or n (%). ^⁎^ *p *< 0.05, ^⁎⁎^ *p *< 0.001 vs healthy controls, ^#^ *p *< 0.05 vs sIL-1R2 ≤median patients.

### 3.2 Soluble IL-1R2 Levels between Groups

**Fig. 3.**
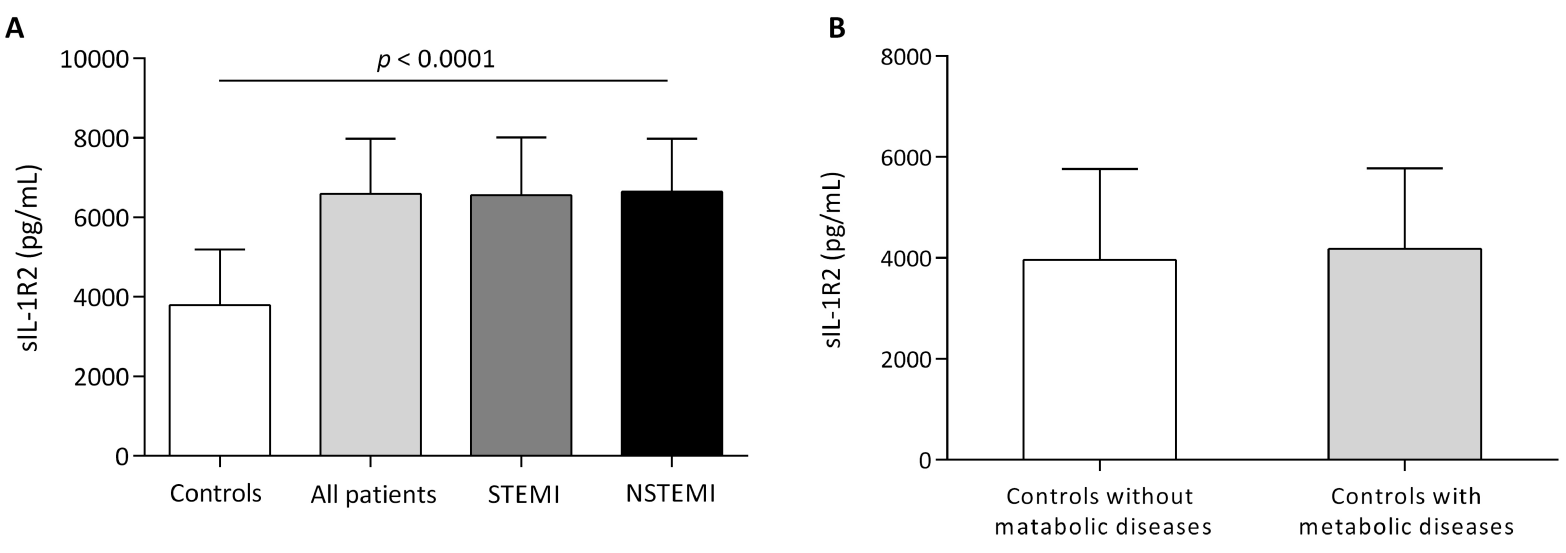
**Circulating levels of sIL-1R2**. (A) Circulating sIL-1R2 levels in AMI patients (104 STEMI, 100 NSTEMI) and controls. (B) Circulating sIL-1R2 levels in controls with (n = 76) and without (n = 128) metabolic disease. Metabolic disease included overweight, obesity, diabetes and metabolic syndrome. Values are medians (interquartile ranges), *p* values for difference between groups of sIL-1R2.

STEMI and NSTEMI populations were characterized by increased levels of sIL-1R2 when compared to healthy controls (Fig. 3A). There were no statistically significant differences in sIL-1R2 between STEMI and NSTEMI patients. To minimize the chance that elevated sIL-1R2 was due to metabolic diseases (overweight/obesity, diabetes and metabolic syndrome), we compared controls with and without metabolic diseases, finding no statistically significant differences (Fig. 3B).

### 3.3 Association between Acute Stage Soluble IL-1R2 Levels and LV Remodeling, LVEF

**Fig. 4.**
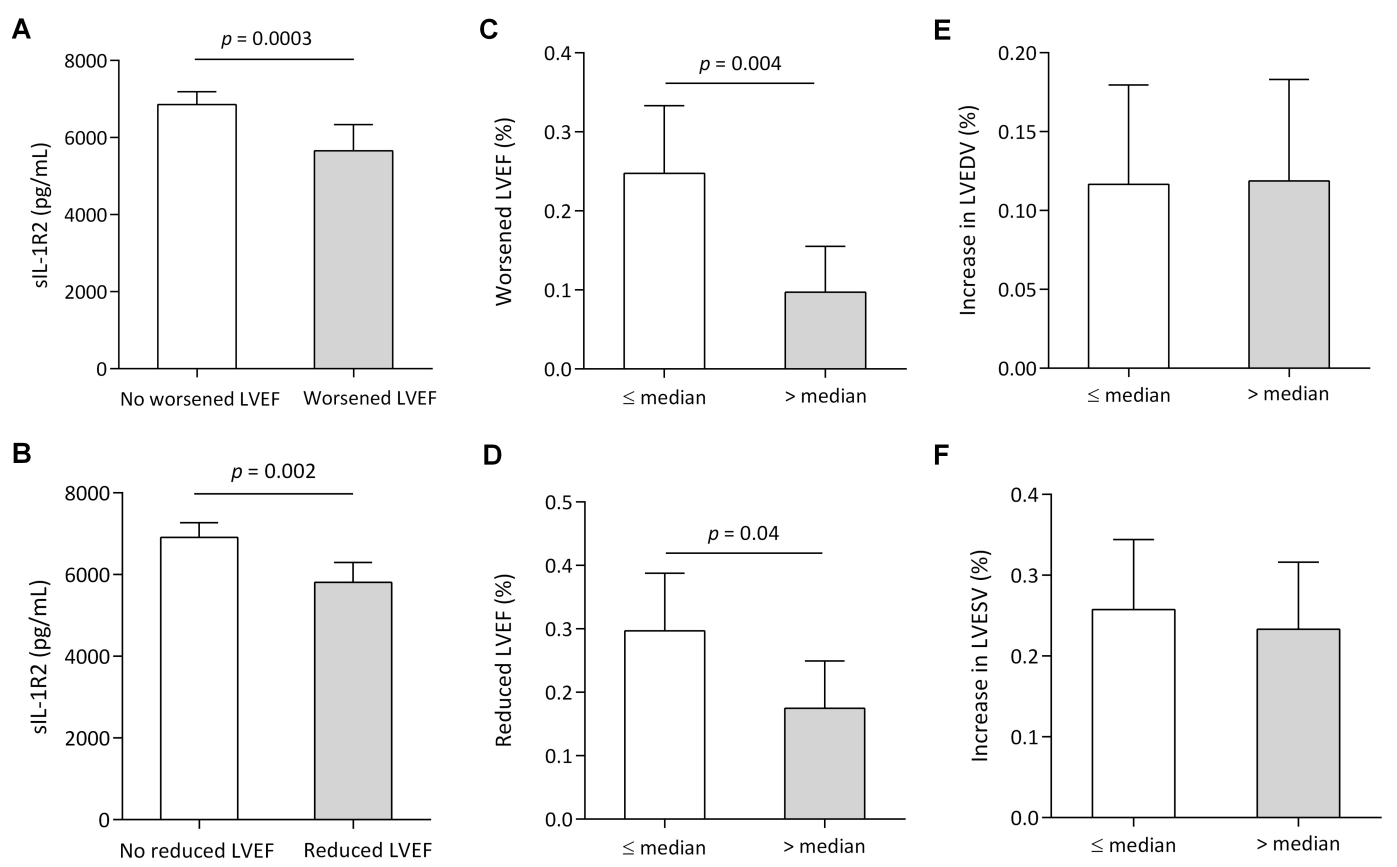
**Myocardial function according to sIL-1R2 levels**. (A) Circulating sIL-1R2 levels in patients with or without worsened LVEF. (B) Circulating sIL-1R2 levels in patients with or without reduced LVEF. (C) Proportion of worsened LVEF (LVEF decreased ˃10%) from the acute stage to 12-month follow-up, according to high or low sIL-1R2 levels: median: 6652.81 pg/mL. (D) Presence of reduced LVEF (final LVEF <50%) after 12 months, according to high or low sIL-1R2 levels. Change in LVEDV (E) and in LVESV (F) from the acute stage to the 12-month follow-up. In A and B, data are presented as medians (interquartile ranges), *p*-values for the differences in sIL-1R2 between groups. In C to F, data are presented as percentages, *p*-values for differences between groups of sIL-1R2.

We then studied the associations between sIL-1R2 and LV remodeling, LVEF. There was no significant association between sIL-1R2 levels and LVEF values determined by echocardiography in the acute phase or at 12-month follow-up (Table 2). However, compared with patients who had worsened LVEF (LVEF decreased ˃10%) or reduced LVEF (final LVEF <50%), sIL-1R2 levels were significantly higher in patients without (Fig. 4A,B). Patients with lower sIL-1R2 levels (less than the median) had a significantly greater incidence of decrease in LVEF ˃10% (Fig. 4C). Moreover, these patients had a higher proportion of reduced LVEF at 12-month follow-up (Fig. 4D), but not adverse LV remodeling, prespecified as a 20% increase in LVEDV (Fig. 4E) or a 15% increase in LVESV (Fig. 4F). In addition, the correlation between sIL-1R2 levels and other inflammation markers were shown in **Supplementary Table 1**. There was a positive correlation of sIL-1R2 levels with neutrophil levels, but not with other inflammatory markers.

**Table 2.** **Myocardial function measured by echocardiography according to low to high sIL-1R2 levels**.

		sIL-1R2 ≤Median	sIL-1R2 ˃Median	*p* value
Acute phase				
	LVEF, %	56.7 (23.0–79.0)	53.4 (21.0–77.0)	0.060
	LVEDV, mL	104.7 (74.2–141.3)	105.8 (73.5–147.4)	0.801
	LVESV, mL	40.2 (27–54.2)	41.1 (28–58.1)	0.462
After 12 months				
	LVEF, %	56.1 (30.0–75.0)	57.1 (23.0–79.0)	0.516
	LVEDV, mL	103.5 (74.0–135.3)	104.0 (74.2–135.3)	0.711
	LVESV, mL	38.7 (26–50.9)	37.3 (24.6–50.9)	0.218

LVEDV, left ventricular end-diastolic volume; LVESV, left ventricular end-systolic volume.

The associations between sIL-1R2 levels and impaired recovery of LV function in AMI patients were investigated using univariate and multivariate logistic regression analyses (Table 3). Low sIL-1R2 levels (less than the median) were associated with increased odds of having worsened LVEF [odds ratio (OR): 3.1, 95% CI: 1.6–8.0, *p* = 0.006]. After adjustment for age, admission NT-proBNP, peak CRP and TnT in multivariable logistic regression analyses, low sIL-1R2 levels remained associated with worsened LVEF after 12 months (OR: 3.7, 95% CI: 1.6–8.5, *p* = 0.002). Patients with low sIL-1R2 levels were more likely to have reduced LVEF (unadjusted OR: 2.0, 95% CI: 1.0–3.9, *p *= 0.041). After adjustment for relevant clinical variables and age, this association remained significant (adjusted OR: 2.1, 95% CI: 1.1–4.3, *p* = 0.035).

**Table 3.** **The associations between sIL-1R2 levels and worsened LVEF and reduced LVEF**.

			Worsened LVEF				Reduced LVEF			
			β	OR	95% CI	*p *value	β	OR	95% CI	*p* value
Univariable analysis										
	Low IL-1R2		1.118	3.1	1.6–8.0	0.006	0.691	2.0	1.0–3.9	0.041
	Age		0.000	1.0	0.97–1.03	0.988	0.029	1.0	1.0–1.1	0.031
	Sex		0.182	1.2	0.5–3.0	0.692	–0.027	0.97	0.5–2.1	0.994
	STEMI		–0.116	0.9	0.4–1.8	0.754	–0.051	0.95	0.5–1.8	0.877
	Triple vessel lesion		–0.739	2.1	0.6–7.4	0.252	–0.143	0.9	0.3–2.3	0.775
	Peak TnT, per SD		0.375	1.5	1.0–2.1	0.034	0.355	1.4	1.0–2.0	0.026
	Admission NT-proBNP, per SD		0.405	1.4	1.1–1.9	0.020	0.399	1.5	1.1–2.0	0.007
	Admission neutrophil, per SD		0.168	1.2	0.8–1.7	0.356	–0.247	0.8	0.5–1.1	0.188
	Peak CRP, per SD		0.393	1.5	1.1–2.0	0.014	0.378	1.5	1.1–2.0	0.013
	NLR, per SD		0.261	1.3	0.9–1.8	0.129	0.058	1.1	0.8–1.5	0.731
	PLR, per SD		0.195	1.2	0.9–1.7	0.245	0.195	1.2	0.9–1.7	0.207
	NPR, per SD		0.280	1.3	0.9–1.8	0.087	0.106	1.1	0.8–1.5	0.506
Multivariable analysis										
	Model 1									
		Low IL-1R2	1.270	3.6	1.5–8.3	0.003	0.778	2.2	1.1–4.5	0.033
		Peak TnT, per SD	0.498	1.7	1.1–2.4	0.011	0.450	1.6	1.1–2.2	0.010
		Admission NT-proBNP, per SD	0.120	1.1	0.8–1.6	0.501	0.337	1.3	0.97–1.8	0.077
		Peak CRP, per SD	0.378	1.5	1.0–2.1	0.029	0.290	1.4	0.99–1.9	0.062
		Age	-̶	-̶	-̶	-̶	0.022	1.0	0.99–1.1	0.126
	Model 2									
		Low IL-1R2	1.302	3.7	1.6–8.5	0.002	0.760	2.1	1.1–4.3	0.035
		Peak TnT, per SD	0.502	1.7	1.1–32.4	0.010	0.441	1.6	1.1–2.2	0.011
		Admission NT-proBNP, per SD	-̶	-̶	-̶	-̶	0.337	1.4	1.0–1.9	0.035
		Peak CRP, per SD	0.403	1.5	1.1–2.1	0.017	0.317	1.4	1.0–1.9	0.048

Worsened LVEF was defined as a decrease in LVEF ˃10%, and reduced LVEF was defined as a final LVEF <50%. Model 1 adjusted for age, admission NT-proBNP, peak CRP and peak TnT. Model 2 adjusted for admission NT-proBNP, peak CRP and peak TnT. LVEF, left ventricular ejection fraction; OR, odds ratio; CI, confidence interval; SD, standard deviation.

#### 3.1.3 Associations between Acute Stage Soluble IL-1R2 Levels and Adverse Clinical Outcomes

**Fig. 5.**
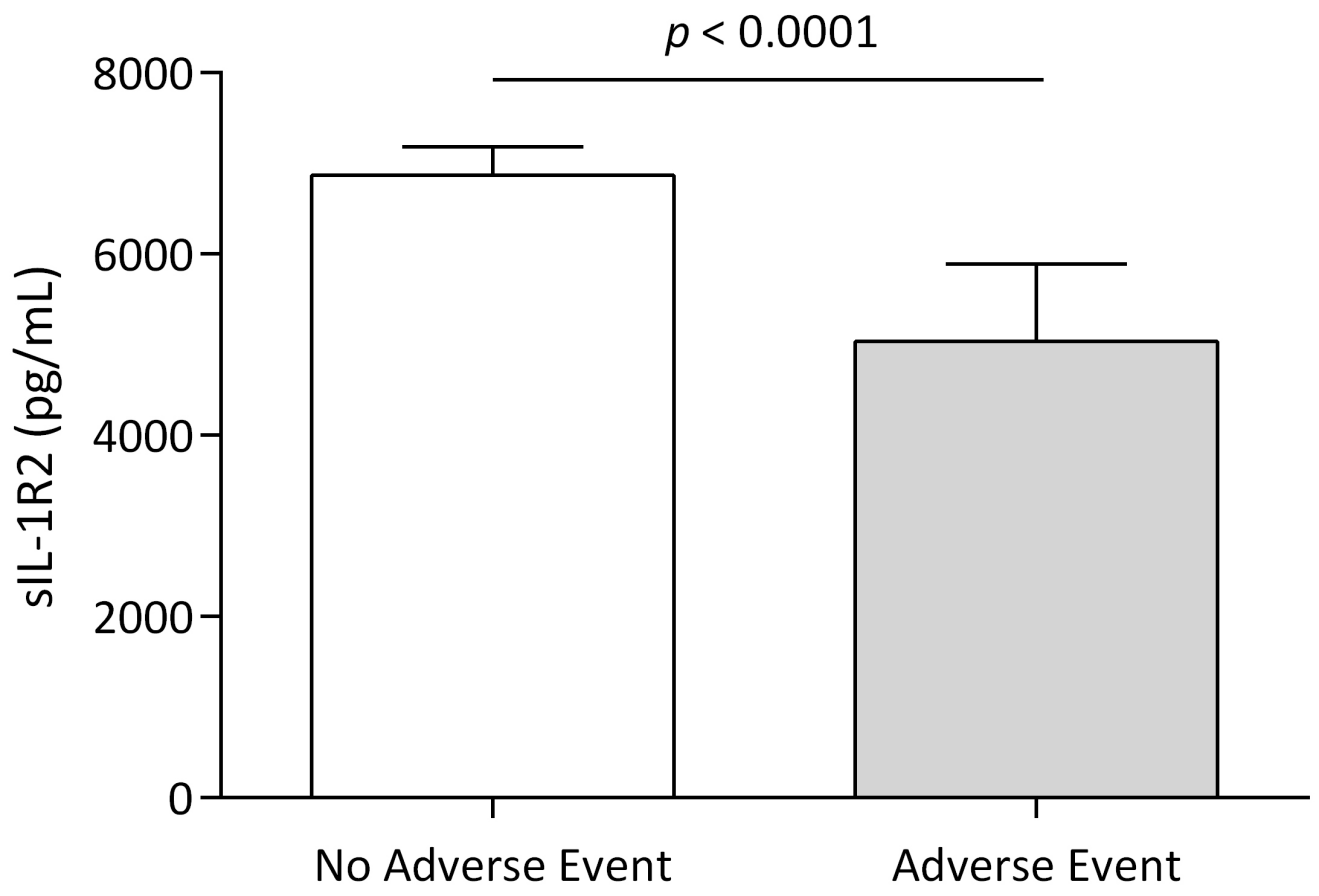
**Adverse clinical events according to sIL-1R2 levels**. Levels of sIL-1R2 in patients with or without adverse clinical events during the first 12 months. Data are presented as medians (interquartile ranges), *p*-values for differences in sIL-1R2 between groups.

**Fig. 6.**
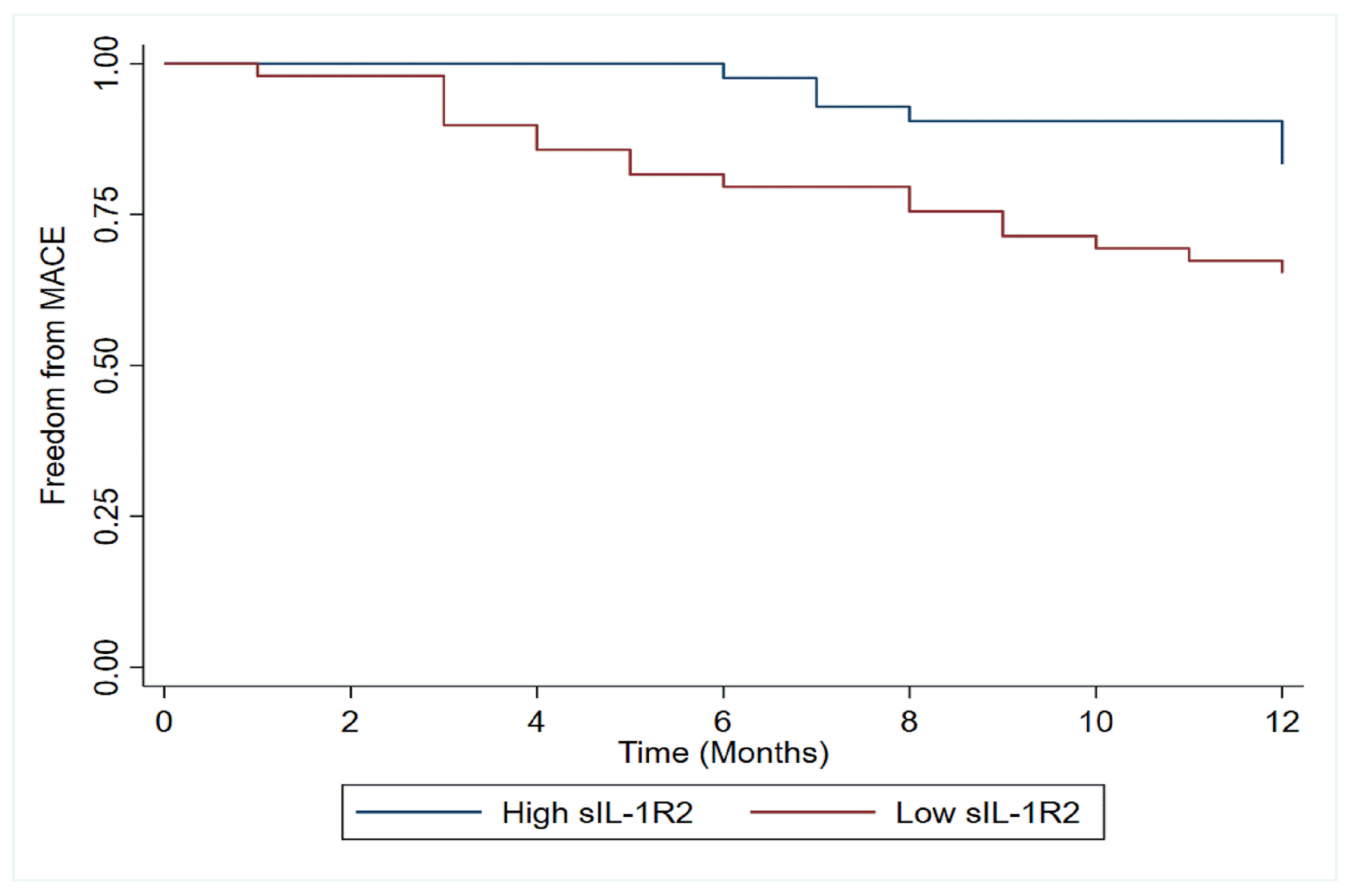
**Kaplan-Meier plots of adverse clinical cardiac events according to sIL-1R2 levels in acute myocardial infarction**. Kaplan-Meier plots of adverse clinical events during the 12-month follow-up according to high or low sIL-1R2. MACE, major adverse cardiac events.

**Fig. 7.**
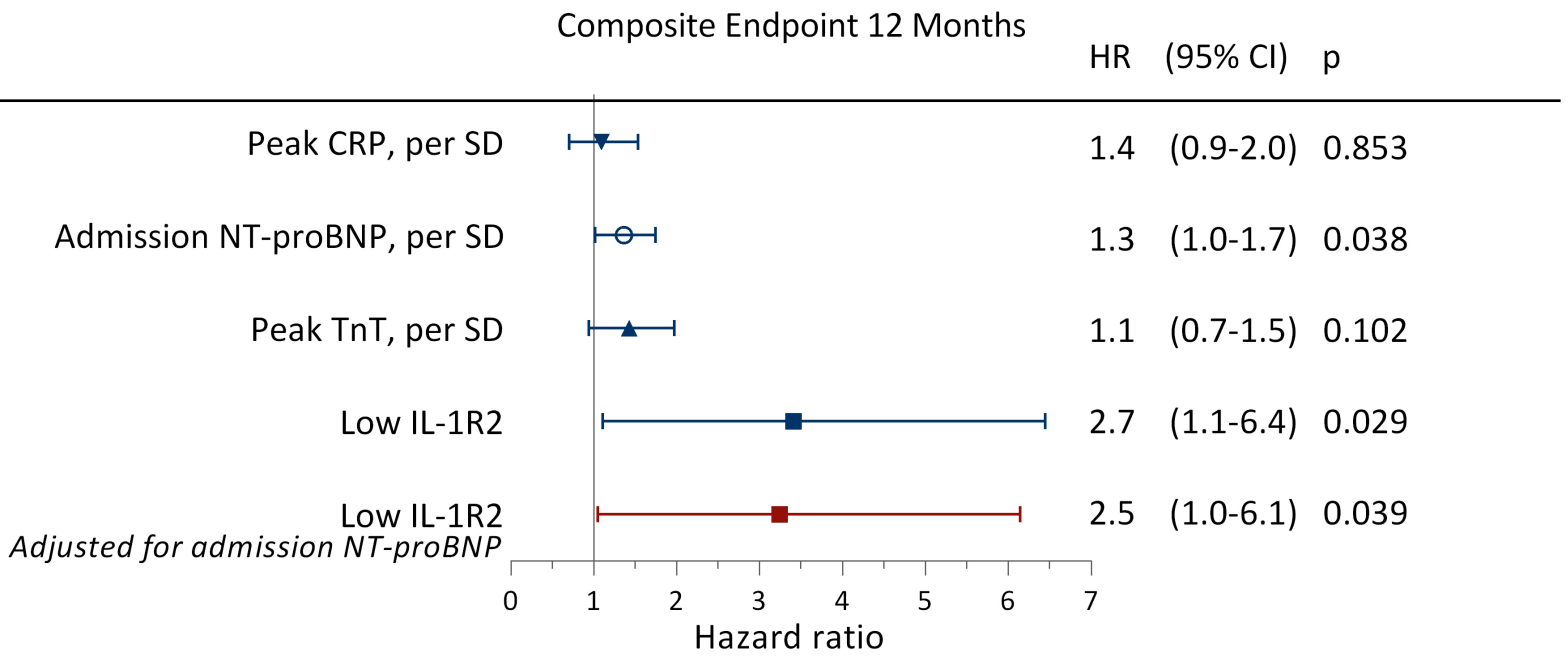
**Hazard ratios for experiencing adverse clinical events**. Unadjusted and adjusted HRs obtained by Cox regression analyses for experiencing an adverse clinical event during the first 12 months of follow-up when having low sIL-1R2 levels (less than the median) during hospitalization. Adverse clinical events were defined as all-cause mortality, reinfarction, rehospitalization for heart failure, or stroke. HR, hazard ratio; CI, confidence interval; other abbreviations as in Fig. 1.

**Fig. 8.**
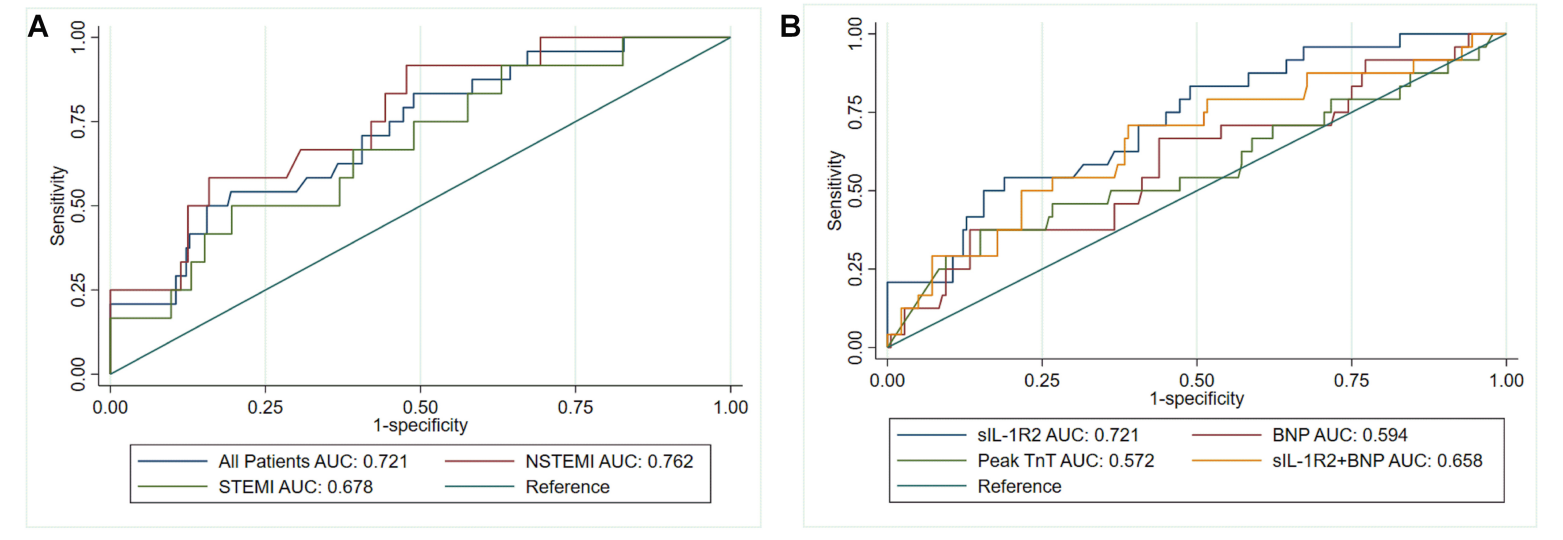
**The discriminative value of sIL-1R2 for adverse clinical events. **(A) ROC curves for an adverse clinical event during the first 12 months after AMI according to sIL-1R2 levels for STEMI, NSTEMI and all patients. (B) ROC curves for detecting an adverse clinical event during the first 12 months after AMI according to sIL-1R2 levels and other biomarkers. Adverse clinical events were defined as all-cause mortality, reinfarction, rehospitalization for heart failure, or stroke. AUC, area under the receiver operating characteristic curve.

During 12 months of follow-up, 24 (11.8%) patients experienced an adverse clinical event (9 reinfarctions, 10 hospitalizations for heart failure, 3 strokes and 2 deaths). In patients who experienced adverse clinical events compared with patients without, sIL-1R2 levels were significantly lower (Fig. 5). Patients with low sIL-1R2 levels had lower freedom from major adverse cardiac events (MACEs) during the first 12 months (Fig. 6). After adjustment for admission NT-proBNP levels, low levels of sIL-1R2 remained associated with an increased risk of experiencing an adverse clinical event during the first 12 months (HR 2.5; 95% CI: 1.0–6.1; *p* = 0.039) (Fig. 7). The ability of sIL-1R2 to discriminate between patients with or without the adverse clinical event was also assessed by the area under the ROC curve, presented in Fig. 8. In all patients, the area under the curve (AUC) was 0.721 (95% CI: 0.617–0.824), and the sIL-1R2 cutoff value of 5022.97 pg/mL had 0.542 sensitivity and 0.806 specificity for detecting AMI. Comparison of AUC between NSTEMI patients and STEMI patients showed that there was an overlap between 95% of the confidence intervals under the ROC curve (*p* = 0.739), suggesting that there was no significant difference in the AUC between the two different groups (Fig. 8A). Soluble IL-1R2 had the highest predictive value for the incidence of an adverse clinical event, with an AUC of 0.721 (95% CI: 0.628–0.896, *p *< 0.0001) when compared with admission BNP (AUC: 0.594, 95% CI: 0.504–0.683, *p* = 0.046) and peak cardiac TnT (AUC: 0.572, 95% CI: 0.474–0.669, *p* = 0.050). Addition of admission BNP slightly impaired the classification of sIL-1R2 between the subject groups (AUC = 0.658, 95% CI: 0.571–0.744, *p* = 0.044) (Fig. 8B).

## 4. Discussion

Circulating levels of sIL-1R2 were elevated in AMI patients as compared to healthy controls. Circulating sIL-1R2 measured early in AMI patients was associated with recovery of LV function and with clinical events during 12 months of follow-up. These findings support the notion that sIL-1R2 may play a crucial role in postinfarction inflammation, and that sIL-1R2 may be clinically useful as a predictor of increased risk of new events in AMI patients.

IL-1 signaling disorders after myocardial infarction can affect infarction healing, cause collateral damage, deteriorate cardiac function and lead to adverse clinical outcomes [18]. IL-1 activity is controlled by the activation of the receptors [4]. Soluble IL-1R2 can competitively bind to IL-1 with high affinity in the circulation and exclude it from the signal transduction complex, so that excessive IL-1 cannot exert its biological function [7]. Moreover, intracellular sIL-1R2 can directly bind to the IL-1β precursor pro-IL-1β, preventing the further conversion of pro-IL-1β into mature IL-1β, inhibiting the IL-1 signaling pathway, and attenuating inflammation [19]. Thus, sIL-1R2 acts as a natural inhibitor of IL-1. However, the role of sIL-1R2 in the inflammatory response accompanying ischemia/reperfusion myocardial damage remains to be explored. Yao *et al*. [20] recently found that the expression of IL-1R2 in AMI patients was higher than that in healthy controls, and a three-gene signature comprising IL1R2, C-C motif chemokine ligand 20 (CCL20), and Intelectin-1 (ITLN1) exhibited outstanding performance in MI diagnosis. Similarly, a previous study demonstrated a persistent increase in sIL-1R2 levels in the acute phase and during 4 months of follow-up in a population of STEMI patients [21]. In the present study, we found that plasma levels of sIL-1R2 were elevated the day after AMI, consistent with previous findings, and suggesting that elevated sIL-1R2 expression might be detected in the circulation and may provide novel therapeutic opportunities for atheroprotection. A previous study demonstrated that elevated sIL-1R2 levels were significantly associated with adverse LV remodeling following STEMI, as assessed as changes in indexed LVEDV and indexed LVESV from the acute phase to 4 months, even after adjustment for relevant clinical variables [21]. Their explanation for the association was that increased sIL-1R2 levels in the acute phase could potentially affect adaptive remodeling induced by IL-1, and thus promote adverse remodeling during follow-up. However, we did not observe this in our study. When comparing our results to those of other studies, it should be noted that there is no unified definition of adverse LV remodeling after AMI [22]. Although sIL-1R2 levels were not associated with adverse LV remodeling outcomes in the present study, the significantly higher frequency of impaired ventricular contractibility after 12 months observed in patients with low sIL-1R2 levels suggests that sIL-1R2 may be involved in their deteriorated LV function. One explanation could be that elevated sIL-1R2 levels represent the activation of a pathway suppressing the activity of IL-1 to protect cardiomyocytes from ischemia/reperfusion injury and to limit the extent of left ventricle remodeling, and that low sIL-1R2 levels may facilitate IL-1 signaling. It is also possible that insufficient sIL-1R2 release promotes atherosclerotic plaque activation and increases the risk of recurrent events. In a previous study, IL-1R2 expression was reduced in monocytes from hyperlipidemic patients and in human atherosclerotic lesions, suggesting a potential role for low IL-1R2 expression in atherosclerosis progression [23]. Although myocardial damage and infarction size are the major determinants of left ventricular remodeling and impaired recovery of LV function, excessive local and systemic inflammation may accelerate this process.

Recently, IL-1R2 has been identified as a pivotal mediator of a broad spectrum of inflammatory cytokines involved in the development of coronary atherosclerosis [24]. In animal models of arthritis [25], IL-1-induced inflammation [26] and cardiac allograft surgery [27], overexpression of IL-1R2 has anti-inflammatory profiles. In transgenic mice, phorbol ester-induced dermal and epidermal inflammation is ameliorated by overexpressing IL-1R2 in the epidermis [28]. The conventional view holds that IL-1R2 is mainly expressed in neutrophils, monocytes and macrophages [5]. Recent research has indicated that there is release of soluble IL-1R2 from injured cardiomyocytes subjected to ischemia/reperfusion conditions. In addition, myocardial ischemia/reperfusion-induced apoptosis is abrogated by IL-1R2 overexpression in cardiomyocytes [29]. Some studies have suggested that IL-1R2 plays a role in regulating monocyte accumulation during myocardial ischemia/reperfusion injury [17]. These findings suggest that IL-1R2 is probably more than redundant in endogenous IL-1 antagonist systems and could be a promising mediator of the inflammatory response in AMI.

Persistent and excessive inflammation unrelated to infarct size has been considered an important contributor to increased risk of ventricular remodeling and adverse clinical events following MI [30]. Abnormal inflammatory status after myocardial infarction is associated with adverse LV remodeling and underlies heart failure pathogenesis [31]. We excluded patients with infectious diseases, chronic inflammatory diseases or cancer to eliminate the effects of other disease processes on the association between low levels of sIL-1R2 and adverse outcomes. The major finding in this study was that low levels of sIL-1R2 during the acute phase of AMI were significantly associated with impaired LV contractibility defined as a decrease in LVEF ˃10% from hospitalization to 12 months and LVEF <50% at 12 months. The association between acute-phase sIL-1R2 levels and poor prognosis remained after adjustment for NT-proBNP, showing that low levels of sIL-1R2 may reflect disadvantageous aspects beyond heart failure itself. Our findings also suggest that IL-1 blockade by sIL-1R2 may have a potential therapeutic effect during the acute phase. This hypothesis should be verified in a future larger cohort of patients with AMI.

Randomized trial data have consistently demonstrated persistent inflammation to be as important a potential therapeutic target for atheroprotection [32, 33]. The CANTOS trial provided proof of concept that attenuating IL-1 inflammation reduces the risk for acute cardiovascular events [8]. Due to the clinical usage of anakinra, the recombinant human IL-1Ra analog, the beneficial effects of IL-1Ra during MI are well documented [34]. In contrast, the role of IL-1R2 in AMI has not been well elucidated. Although anakinra is a valuable therapeutic tool, it has a short *in vivo* half-life, necessitating daily injection [35]. IL-1R2 has a longer half-life, low affinity for IL-1Ra and high affinity for IL-1β, and thus may be a promising therapeutic candidate [36]. In the present study, the association between low sIL-1R2 and adverse clinical outcomes enhances the likelihood of a therapeutic potential of targeting sIL-1R2 in AMI, and will warrant being more thoroughly addressed in future studies.

## 5. Study Limitations

The limitations of this study should be acknowledged. The results of this study provide no evidence of a causal relationship involving sIL-1R2 and LV function or adverse clinical outcomes. The reported number of adverse events in the present cohort was relatively small. Moreover, we used echocardiography to assess adverse remodeling in the present study, which needs to be considered when interpreting the results. Furthermore, we lacked follow up measurements and the temporal profile of sIL-1R2 on the AMI patients between days 1 and 12 months. Other inflammatory markers, such as IL-1α, IL-1β, IL-1R1, and IL-1RAcP, which might reflect the inflammatory status more accurately, were not evaluated in the study. Future studies, including experimental studies, are necessary to further evaluate the role of sIL-1R2 in AMI. Nonetheless, our data demonstrate that sIL-1R2 could be an unrecognized mediator of recovery of LV function in AMI patients.

## 6. Conclusions

The present study demonstrated that low levels of sIL-1R2 in the acute phase of AMI patients were associated with impaired recovery of LV function and increased future adverse clinical events. The results indicated that sIL-1R2 may be a clinically useful biomarker for risk prediction in AMI patients, and sIL-1R2 itself may be a novel target for atherothrombotic protection.

## Supplementary Material

Supplementary material associated with this article can be found, in the online version, at https://doi.org/10.31083/j.rcm2311372.
