# Supplementary material for: Association of Soluble IL-1 Receptor Type 2 with Recovery of Left Ventricular Function and Clinical Outcomes in Acute Myocardial Infarction
Source: Rev Cardiovasc Med. 2022 Oct 31;23(11):372. doi: 10.31083/j.rcm2311372 (PMC11269066; doi:10.31083/j.rcm2311372)
Supplement: Supplementary file 1 [file 2153-8174-23-11-372-s1.docx]

Supplementary Table 1. Correlation analysis of sIL-1R2 levels with other inflammation markers.

|  | sIL-1R2 | |
| --- | --- | --- |
| Inflammation markers | r | *p* |
| WBC | 0.167 | 0.017 |
| Neutrophil | 0.303 | < 0.01 |
| NLR | 0.105 | 0.153 |
| PLR | -0.015 | 0.827 |
| NPR | -0.080 | 0.273 |
| CRP | 0.068 | 0.331 |

WBC, white blood cell; NLR, neutrophil-to-lymphocyte ratio; PLR, platelet-to-lymphocyte ratio; NPR, neutrophil-to-platelet ratio; CRP, C-reactive protein.
